# Incidence, Mechanisms of Injury and Mortality of Severe Traumatic Brain Injury: An Observational Population-Based Cohort Study from New Zealand and Norway

**DOI:** 10.1007/s00268-022-06721-8

**Published:** 2022-09-05

**Authors:** Clemens Weber, Joakim Stray Andreassen, Siobhan Isles, Kenneth Thorsen, Paul McBride, Kjetil Søreide, Ian Civil

**Affiliations:** 1 Department of Neurosurgery, Stavanger University Hospital, PO Box 8100, 4068 Stavanger, Norway; 2 Department of Quality and Health Technology, University of Stavanger, Stavanger, Norway; 3 Department of Neurosurgery, St. Olavs Hospital, Trondheim, Norway; 4 New Zealand Trauma Network, Wellington, New Zealand; 5 Department of Surgery, Stavanger University Hospital, Stavanger, Norway; 6 Section of Traumatology, Stavanger University Hospital, Stavanger, Norway; 7 Department of Clinical Medicine, University of Bergen, Bergen, Norway; 8 Department of Surgery, Auckland City Hospital, Auckland, New Zealand

## Abstract

**Background:**

Comparing trauma registry data from different countries can help to identify possible differences in epidemiology, which may help to improve the care of trauma patients.

**Methods:**

This study directly compares the incidence, mechanisms of injuries and mortality of severe TBI based on population-based data from the two national trauma registries from New Zealand and Norway. All patients prospectively registered with severe TBI in either of the national registries for the 4-year study period were included. Patient and injury variables were described and age-adjusted incidence and mortality rates were calculated.

**Results:**

A total of 1378 trauma patients were identified of whom 751 (54.5%) from New Zealand and 627 (45.5%) from Norway. The patient cohort from New Zealand was significantly younger (median 32 versus 53 years; *p* < 0.001) and more patients from New Zealand were injured in road traffic crashes (37% versus 13%; *p* < 0.001). The age-adjusted incidence rate of severe TBI was 3.8 per 100,000 in New Zealand and 2.9 per 100,000 in Norway. The age-adjusted mortality rates were 1.5 per 100,000 in New Zealand and 1.2 per 100,000 in Norway. The fatality rates were 38.5% in New Zealand and 34.2% in Norway (*p* = 0.112).

**Conclusions:**

Road traffic crashes in younger patients were more common in New Zealand whereas falls in elderly patients were the main cause for severe TBI in Norway. The age-adjusted incidence and mortality rates of severe TBI among trauma patients are similar in New Zealand and Norway. The fatality rates of severe TBI are still considerable with more than one third of patients dying.

## Introduction

Traumatic brain injury (TBI) is a major cause of mortality and morbidity and one of the main challenges in modern trauma care [1]. Severe TBI is known to be the major cause of death in trauma patients with a huge impact on patients, families and society [2, 3]. TBI management has been identified as one of the main priority areas in trauma research [4].

**Fig. 1:**
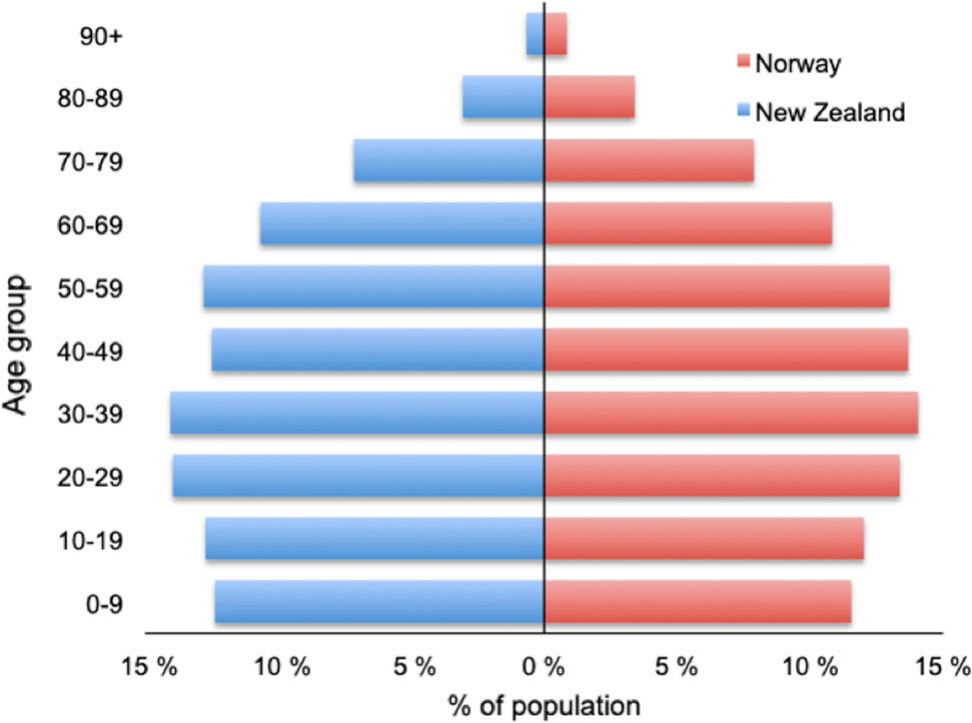
Age demographics in New Zealand and Norway as per December 2020 (*Source*: Stats NZ, Statistics Norway)

New Zealand and Norway are similar in size, geography, population, life expectancy (Table 1) and age demographics (Fig. 1). Both countries have established national trauma registries to prospectively collect population-based data representing valuable sources of information on trauma patients [5, 6]. Both countries also face similar challenges, such as long transportation times due to their geography, but they have a similar standard of living and health care systems with publicly funded trauma care. Comparing registry data from different countries with otherwise similar healthcare environments can help to identify possible differences in epidemiology, treatment and outcome which again may help to improve the care of trauma patients. However, one of the main problems in comparing data from different registries may be variation in data collection and therefore a validation of the collected data is necessary [7].

**Table 1:** Comparison of population, size and life expectancy in New Zealand and Norway

Variable	New Zealand	Norway
Population	5,106,400	5,367,580
Population density	18 per km^2^	15 per km^2^
Urban population	86.9%	83.4%
Size in km^2^	263,310	365,268
Median age in years	37.6	39.8
Life expectancy in years	82.8	82.9
Female	83.9	84.8
Male	80.3	81.5
Largest city	Auckland	Oslo
Population	417,910	690,400

Data as per December 2020 (Source: Stats NZ, Statistics Norway)

The aim of this study was to directly compare the incidence, injury mechanisms and outcome of severe TBI based on population-based data from the two national trauma registries from New Zealand and Norway.

## Material and methods

### Ethics

The Regional Committee for Medical and Health Research Ethics of Western Norway approved this observational study (REK143902/2020). The study protocol has been approved by the Data Governance Group of the New Zealand Trauma Registry and the Advisory Board of the Norwegian Trauma Registry.

### Study design and period

This observational registry-based study reports data according to the standards for observational research (STROBE guidelines) [8]. The study uses prospectively collected data as part of the existing national trauma registries of New Zealand and Norway. The study period includes all patients with severe TBI included in both registries between 01.01.2017 and 31.12.2020.

### Trauma registry data and data completeness

The New Zealand Trauma Registry collects data from 22 hospitals delivering trauma care in New Zealand providing national, population-based data on trauma patients. The Norwegian National Trauma Registry collects data from 38 hospitals admitting trauma patients. Data completeness was checked and was high in both trauma registries. The New Zealand Trauma Registry had a data completeness of 100% for patient and injury characteristics as well as outcome variables. The Norwegian Trauma Registry had a data completeness of 98.7% for patient age, 99.7% for type, mechanism and intention of injury and 100% for sex, as well as 99.7% for all outcome variables.

### Study population

This current study included all patients registered with severe TBI in either of the national registries for the 4-year study period who were transported to hospital by road or air ambulance. There are a variety of definitions for severe TBI in the literature [9–11]. To identify only patients with severe TBI and exclude patients with unconsciousness due to other causes (i.e. stroke, intoxication, medical cause, etc.) patients were identified by the following criteria: Glasgow Coma Scale (GCS) score between 3 and 8 at scene of injury, Abbreviated Injury Scale (AIS) severity score for head injuries of 3 or more and Injury Severity Score (ISS) of 13 or more [12]. Hence, GCS (3–8), AIS head injury (3 or more) and ISS (13 or more) had to be present in order to be included.

### Epidemiology

In order to compare the data between the two national trauma registries the background population-based statistics was retrieved from the official national data agencies of both countries, Statistics Norway (www.ssb.no) and Stats NZ (www.stats.govt.nz). For both countries the estimated resident populations for 2017–2020 by 5-year age group were extracted.

### Outcome

The main outcome parameters of this study were the incidence and mortality rates per 100,000 people in New Zealand and Norway. Fatality rates (in %) after discharge from acute care at the definitive care hospital (e.g. receiving hospital and/or hospital with neurosurgical care) and discharge destination from acute care (home, rehabilitation, other hospital ward, other destination) were other outcome measures. All outcome measures were compared between the two national registries.

### Statistical analysis

All statistical analyses were performed with SPSS version 26.0 (IBM, 2019) except direct standardization, which was performed with R version 4.1.1 (R Core Team, 2021). Patient demographics, injury, treatment and outcome data were described using descriptive statistics, using medians and interquartile range (IQR) where applicable for continuous variables, and rates for categories. Age-adjusted incidence and mortality rates were calculated using direct standardization, with confidence intervals calculated following Dobson et al.’s method [13]. The Norwegian standard population in 2017 was used as the reference population. Adjustments were made by the following age groups: 0–4, 5–9, 10–14, 15–19, 20–24, 25–34, 35–44, 45–54, 55–64, 65–74, 74–84, 85–89, 90 + years. The chi-square test was used to analyze categorical variables and rates. The independent samples median test was used for continuous variables. All tests were two-sided, statistical significance was defined as *p* < 0.050.

## Results

During the 4-year study period, a total of 1378 trauma patients with severe TBI were identified according to the inclusion criteria from the national trauma registries of New Zealand and Norway of which 751 (54.5%) from New Zealand and 627 (45.5%) from Norway.

### Age-adjusted incidence rate and demography

**Fig. 2:**
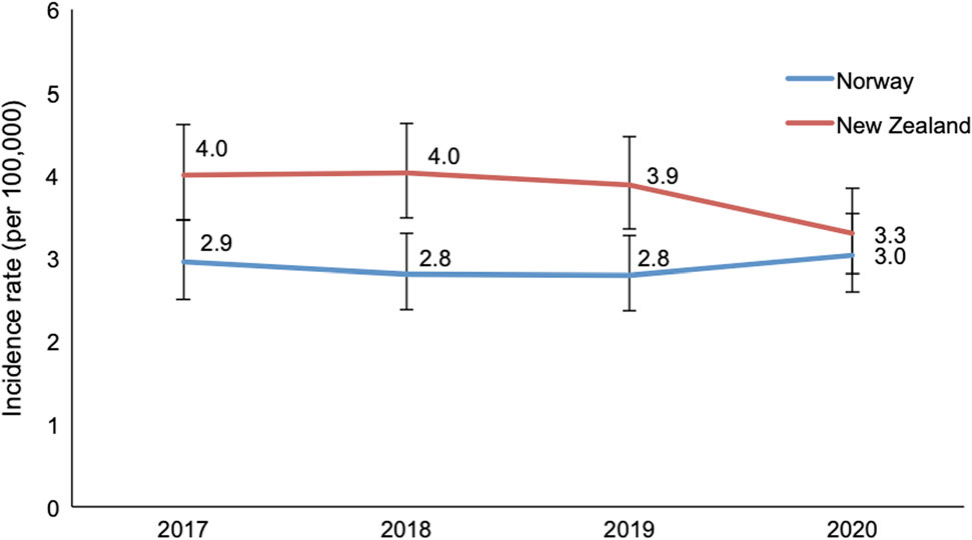
Age-adjusted incidence rate of severe TBI per 100,000 population in New Zealand and Norway, 2017–2020. Error bars are 95% confidence intervals of direct standardisation using Dobson et al.’s method

**Fig. 3:**
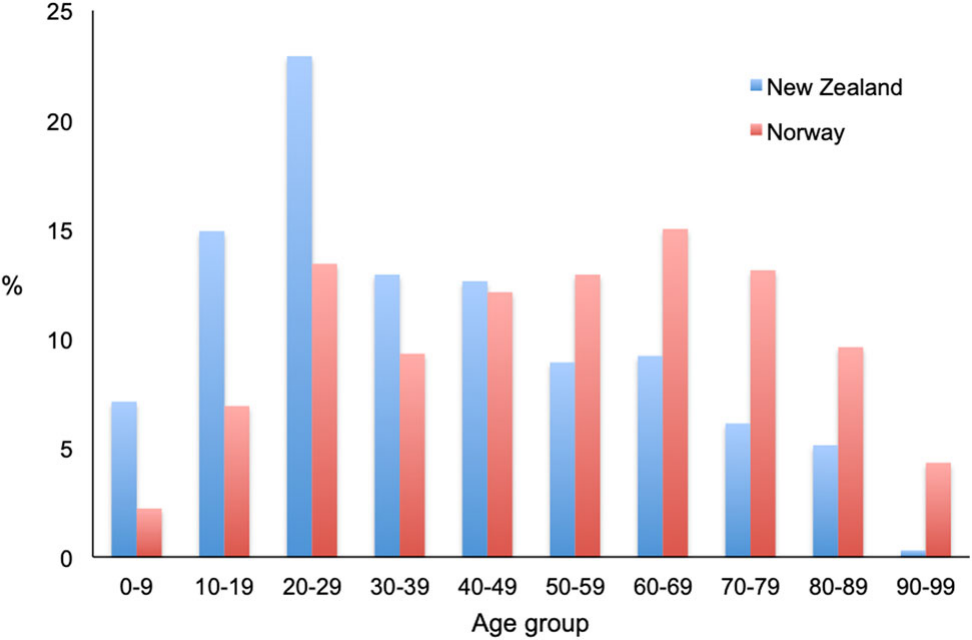
Age group distribution for patients with severe TBI in New Zealand and Norway (in %), 2017–2020

**Fig. 4:**
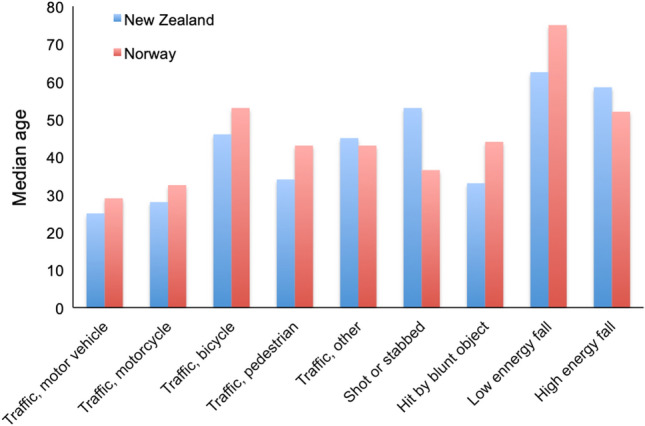
Median age and mechanism of injury for patients with severe TBI in New Zealand and Norway, 2017–2020

The age-adjusted incidence rate of severe TBI was 3.8 patients per 100,000 inhabitants in New Zealand and 2.9 per 100,000 in Norway. The age-adjusted incidence rates per year are presented in Fig. 2. Patient and injury variables are presented in Table 2. The Norwegian cohort consisted of significantly older patients (Table 2); the distribution among age groups is presented in Fig. 3. There was no difference in sex distribution between both cohorts (Table 2). The median age of patients in groups with the different mechanisms of injury are presented in Fig. 4. Patients under the age of 65 years had lower median ISS in New Zealand (26.0; IQR 22.0–34.0) compared to Norway (29.0; IQR 25.0–38.0). Above the age of 65 years, patients in New Zealand (26.0; IQR 25.0–30.0) had the same median ISS compared to patients from Norway (26.0; IQR 22.5–29.0).

**Table 2:** Patient and injury characteristics after severe TBI in New Zealand and Norway, 2017–2020

Variable	New Zealand (*n* = 751)	Norway (*n* = 627)	*p*-value
Age in years, median (IQR)	32 (21–55)	53 (32–71)	< 0.001
*Sex, no. (%)*			
Male	543 (72%)	463 (74%)	
Female	208 (28%)	164 (26%)	0.522
*Dominating type of injury, no. (%)*			
Blunt	732 (97%)	614 (98%)	0.578
Penetrating	19 (3%)	11 (2%)	0.325
*Mechanism of injury, no. (%)*			
Traffic, motor vehicle	281 (37%)	79 (13%)	< 0.001
Traffic, motorcycle	55 (7%)	26 (4%)	0.013
Traffic, bicycle	33 (4%)	50 (8%)	0.005
Traffic, pedestrian	67 (9%)	23 (4%)	< 0.001
Traffic, other	13 (2%)	20 (3%)	0.078
Shot or stabbed	19 (3%)	12 (2%)	0.442
Hit by blunt object	91 (12%)	47 (7%)	0.004
Low energy fall	82 (11%)	148 (24%)	< 0.001
High energy fall	98 (13%)	201 (32%)	< 0.001
*Intention of injury, no. (%)*			
Unintentional	633 (84%)	543 (87%)	0.227
Self-inflicted	33 (4%)	47 (7%)	0.014
Assault	75 (10%)	20 (3%)	< 0.001

*IQR*, Interquartile range

### Mortality and outcome

**Fig. 5:**
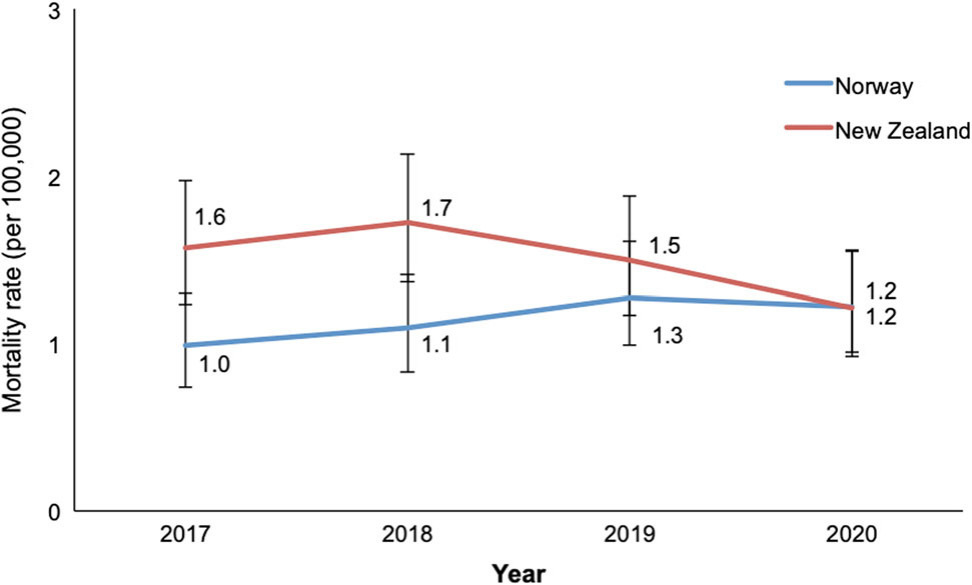
Age-adjusted mortality rate of severe TBI per 100.000 inhabitants in New Zealand and Norway, 2017–2020. Error bars are 95% confidence intervals of direct standardization using Dobson et al.’s method

**Fig. 6:**
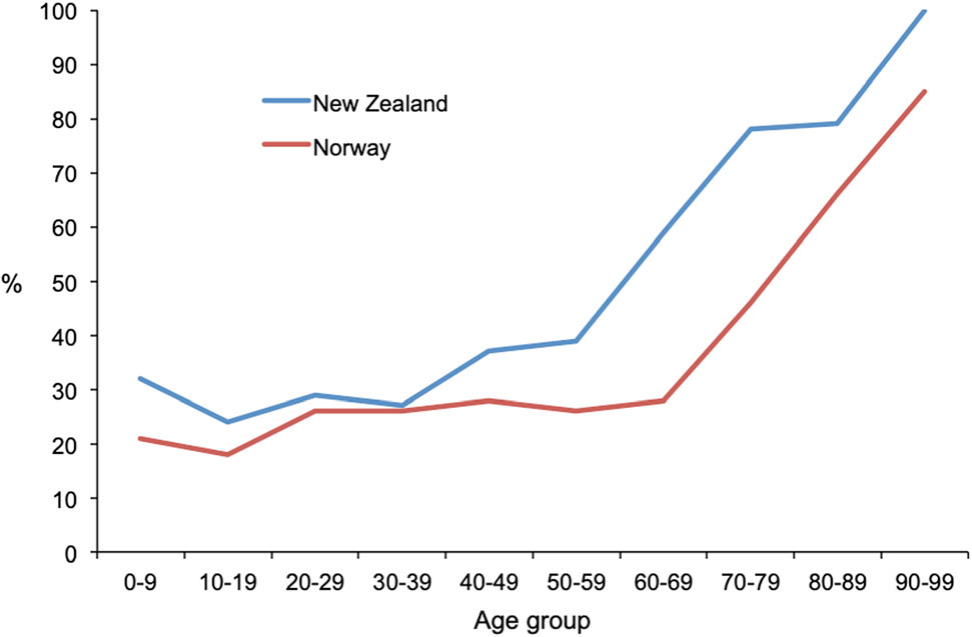
Fatality rate in % per age group for patients with severe TBI in New Zealand and Norway, 2017–2020

The age-adjusted mortality rate due to severe TBI was 1.5 per 100,000 in New Zealand and 1.2 per 100,000 in Norway. The age-adjusted mortality rates per year are presented in Fig. 5. The percentage of fatal outcome due to severe TBI in relation to number of patients did not differ significantly between the two cohorts (Table 3). Fatality rates according to age groups are presented in Fig. 6. In children under the age of 18 years 32 out of 121 (26.4%) died due to severe TBI in New Zealand, where the number was 6 out of 40 (15.0%) in Norway (*p* = 0.140). In patients < 50 years of age there was no difference in fatality between the national cohorts (154 of 375 (29.1%) in New Zealand vs. 74 of 275 (26.9%) in Norway; *p* = 0.548). In patients with severe TBI ≥ 50 years, a significantly higher number of patients died in New Zealand compared to Norway (135 of 222 (60.8%) vs. 172 of 342 (50.3%); *p* = 0.023) and in patients above 65 years of age the difference was even larger (82 of 109 (75.2%) vs. 111 of 205 (54.1%); *p* = 0.001).

**Table 3:** Outcome data with survival status and discharge destination of the surviving patients after severe TBI in New Zealand and Norway, 2017–2020

Variable	New Zealand	Norway	*p*-value
*Survival status at discharge, no. (%)*			
Alive	462 (61.5%)	413 (65.5%)	0.112
Dead	289 (38.5%)	216 (34.2%)	
*Discharge destination, no. (%)*			
Home	67 (14.5%)	62 (15.0%)	0.823
Rehabilitation	301 (65.2%)	132 (32.0%)	< 0.001
Other hospital ward	52 (11.3%)	175 (42.4%)	< 0.001
Other	42 (9.1%)	40 (9.7%)	0.764

*IQR*, Interquartile range

In New Zealand, significantly more patients with severe TBI were transferred directly to a rehabilitation unit after primary care versus Norway (301 of 462 (65.2%) vs. 132 of 413 (32%); *p* < 0.001). In Norway, more patients were transferred to another hospital ward (175 of 413 (42.2%) vs. 52 of 462 (11.3%); *p* < 0.001). In both countries a similar number of patients were directly discharged to home care. The distribution of discharge destinations for both cohorts is presented in Table 3.

## Discussion

This bi-national, observational study of trauma patients with severe TBI showed similar age-adjusted incidence rates of severe TBI in New Zealand and Norway for the 4-year study period. Between 3 and 4 patients per 100,000 inhabitants suffered from severe TBI in both countries each year. The results showed also similar age-adjusted mortality rates in New Zealand and Norway, between 1.0 and 1.7 patients per 100,000 inhabitants died in both countries in each year of the study period. Notably, the fatality of severe TBI in trauma patients was still high with more than one third of admitted patients not surviving this injury. This is the first study comparing contemporary data from two mature trauma systems, presenting reliable data on real-life incidence and mortality rates of severe TBI in trauma patients.

### Age-adjusted incidence rate, demography and injury mechanisms

There are few studies reporting age-adjusted incidence rates of TBI and most of them are based on institutional or regional data and not based on national data. A Norwegian study on patients with severe TBI (defined by GCS 8 or lower) reported an age-adjusted incidence rate of 5 patients per 100,000 inhabitants in 2012; however, the data were retrieved from the Norwegian trauma centers and patients that were treated or died at other hospitals were not included, therefore the actual incidence rate might have been higher at that time [14]. Systematic reviews have reported incidence rates of severe TBI between 7 and 17 per 100,000 inhabitants [15, 16]. Our results showed lower incidence rates of severe TBI in Norway and New Zealand in the recent years. However, other authors have used different definitions of TBI severity, which makes direct comparison difficult.

The median age of trauma patients with severe TBI was significantly higher in Norway compared to New Zealand. This might be influenced by underreporting of elderly patients with severe TBI who are at risk for under-triage [17]. A recent study comparing data from Australia and Europe also showed a younger patient age in the Australian cohort with a median age of 32 years and similar to the patients from New Zealand presented here, whereas the European patients were still younger (median age of 44 years) than the Norwegian cohort in our study (median age of 53 years) [18].

The patients’ median age and sex distribution are also reflected by the mechanism of injury; in New Zealand significantly more patients were injured in road traffic crashes which are more common in younger adult males [19]. In recent years there has been an increase in road crashes in New Zealand probably leading to a higher number of young, male patients suffering from severe TBI [20]. In Norway increased focus on road traffic safety has reduced the number of road traffic fatalities in 2020 compared to data from New Zealand [21, 22]. A recent WHO report on road safety states that road traffic crashes cause almost 1.5 million deaths annually, making it the major cause of death for younger people [23]. Norwegian authorities established a National Action Plan for Road Safety in 2002, which is updated regularly [24]. The aim is to improve road traffic safety in Norway based on a vision of zero fatalities and severe road traffic related injuries. The number of road traffic fatalities per 100,000 inhabitants in Norway has fallen by 73% between 2000 and 2019 [21]. The Ministry of Transport of New Zealand has recently established a strategy with the vision that no one is killed or seriously injured in road traffic crashes [25]. Intensifying preventive measures to improve road traffic safety could help to further reduce the number of severely injured TBI.

Patients with severe TBI due to low energy falls were older in Norway with a median age of 75 years compared to a median of 62.5 years in New Zealand. Low energy falls leading to head injuries are the most frequent cause of severe injury in elderly patients in Norway [6, 26]. With an increasing and more active elderly population the number of elderly patients with severe TBI is likely to increase in the future. Prevention of falls in the elderly could help to reduce the number of severe TBI in this patient group [27]. In pediatric patients falls and road traffic crashes are the most common injury mechanisms for severe TBI and prevention should also be the main target to reduce numbers for this age group [28].

### Mortality and outcome

The age-adjusted mortality rate of severe TBI did not differ much between New Zealand and Norway. The literature reporting age-adjusted, population-based mortality rates for severe TBI is scarce. A systematic review from 2006 based on European studies reported an average mortality of TBI of 15/100,000 [29]. A recent review reported crude mortality rates ranging from 9 to 28 per 100,000 inhabitants per year based on country-level studies [15]. Thus, Norway and New Zealand have presumably very low mortality rates compared to other countries.

The overall fatality rate due to severe TBI did not differ significantly between New Zealand and Norway. The Norwegian cohort had lower fatality rates in all age groups, but the larger number of older patients with severe TBI in Norway resulted in a similar overall fatality rate for both cohorts. The fatality rate in pediatric patients was lower in Norway and younger patient age has earlier been identified as a predictor for survival after severe TBI [30]. The most common injury patterns in younger patients are road traffic crashes and falls. Therefore prevention should focus on improved traffic safety and reducing falls.

In older patients with severe TBI more than 50% had a fatal outcome due to this condition in Norway, while this number was even higher in New Zealand (74%). Recent studies based on regional registries from Oslo, Norway, and Victoria, Australia reported similar findings [31, 32]. A large meta-analysis reported a fatality rate of 65% for severe TBI patients over 60 years of age [33]. Elderly trauma patients are at greater risk of under-triage and most under-triage deaths are secondary to TBI [34–36]. Under-triage has been reported for elderly patients, who are less often triaged with the highest priority level. Also, longer time from admission to first CT scan has been reported for older patients [17]. Certainly there are several reasons such as co-morbidity and age, but under-triage may be one of the factors influencing the higher mortality among elderly patients [37].

### Strengths and limitations

One of the main strengths of this study is the prospectively collected, population-based data from national registries with very little missing data. Both countries have universal health coverage with valid population statistics. Hence, the data may be viewed as reliable for contemporary outcomes in severe TBI.

In this study, a strict definition of TBI severity was used to exclude patients with other conditions that might mimic severe TBI. A potential limitation of this study would be the comparability to other studies using different definitions of severe TBI.

Another limitation is the lack of long-term outcome data. Many TBI patients are likely to improve after long-term rehabilitation treatment.

## Conclusions

The age-adjusted incidence and mortality rates of severe TBI among trauma patients are similar in New Zealand and Norway. The fatality rates of severe TBI are still considerable with more than one third of patients dying. Road traffic crashes in younger patients and falls in elderly patients are the main causes for severe TBI in both countries. Preventive measures such as improved road traffic safety and reducing the risk of falls can help to reduce the number of patients suffering severe TBI.
